# Supplementary material for: Vimentin intermediate filaments stabilize dynamic microtubules by direct interactions
Source: Nat Commun. 2021 Jun 18;12:3799. doi: 10.1038/s41467-021-23523-z (PMC8213705; doi:10.1038/s41467-021-23523-z)
Supplement: Supplementary file 3 — Description of Additional Supplementary Files [file 41467_2021_23523_MOESM3_ESM.docx]

**Description of Additional Supplementary Files**

File Name: Movie 1

Description: Confocal fluorescence movie of an IF-microtubule pair. We moved the IF vertically while it was in a perpendicular orientation with respect to the microtubule. The microtubule-IF pair interacted strongly; the microtubule broke off a bead. Scale bar 5 μm and 4.4 s per frame.

File Name: Movie 2

Description: Confocal fluorescence movie of an IF-microtubule pair. We moved the IF at a 45° angle with respect to the microtubule. At first the filaments did not interact, then interactions became apparent by the deformation of the microtubule. At the end of the movie the microtubule broke off a bead due to strong interactions. Scale bar 5 μm and 2.4 s per frame.

File Name: Movie 3

Description: Confocal fluorescence movie of an IF-microtubule pair. We moved the IF horizontally while it was in a perpendicular orientation with respect to the microtubule. The IF deformed while interacting with the microtubule. Eventually the interactions were so strong that the microtubule broke off the beads.

Scale bar 5 μm and 2.2 s per frame.

File Name: Movie 4

Description: Confocal fluorescence movie of an IF-microtubule pair. We moved the IF vertically while it was in a perpendicular orientation with respect to the microtubule. The IF-microtubule pair interacted, leading to deformations of the microtubule. Eventually the IF broke. Scale bar 5 μm and 5.6 s per frame.

File Name: Movie 5

Description: Confocal fluorescence movie of an IF-microtubule pair. We moved the IF vertically while it was in a perpendicular orientation with respect to the microtubule. The IF-microtubule did not interact. Scale bar 5 μm and 2.5 s per frame.

File Name: Movie 6

Description: (same data as in Supplementary Figure 5): A strong IF-microtubule interaction that persisted for downwards and upward pulling direction. The IF was first moved downward until the microtubule broke off the right-hand-side bead. When the IF was moved upward, the microtubule re-attached to the right-hand-side bead and then broke off again, showing that the IF-microtubule interaction persisted even when pulling in different directions. Scale bar 5 μm and 8.4 s per frame.
