# Supplementary figures and images for: Vimentin intermediate filaments stabilize dynamic microtubules by direct interactions
Source: Nat Commun. 2021 Jun 18;12:3799. doi: 10.1038/s41467-021-23523-z (PMC8213705; doi:10.1038/s41467-021-23523-z)

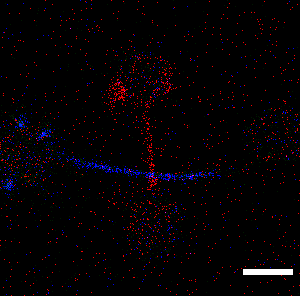

Supplement: Supplementary file 4 — video 1 [file 41467_2021_23523_MOESM4_ESM.gif]

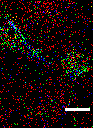

Supplement: Supplementary file 5 — video 2 [file 41467_2021_23523_MOESM5_ESM.gif]

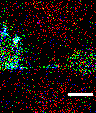

Supplement: Supplementary file 6 — video 3 [file 41467_2021_23523_MOESM6_ESM.gif]

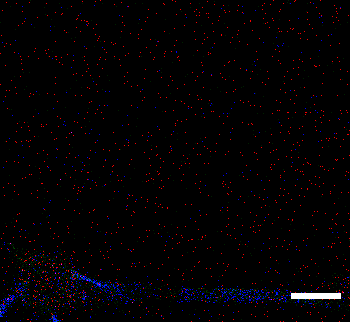

Supplement: Supplementary file 7 — video 4 [file 41467_2021_23523_MOESM7_ESM.gif]

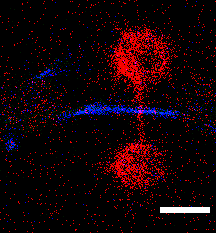

Supplement: Supplementary file 8 — video 5 [file 41467_2021_23523_MOESM8_ESM.gif]

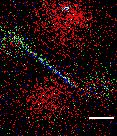

Supplement: Supplementary file 9 — video 6 [file 41467_2021_23523_MOESM9_ESM.gif]
