# Supplementary material for: Vimentin intermediate filaments stabilize dynamic microtubules by direct interactions
Source: Nat Commun. 2021 Jun 18;12:3799. doi: 10.1038/s41467-021-23523-z (PMC8213705; doi:10.1038/s41467-021-23523-z)
Supplement: Supplementary file 10 — Reporting Summary [file 41467_2021_23523_MOESM10_ESM.pdf]

nature research

Corresponding author(s): Prof Sarah Köster

Last updated by author(s): Mar 23, 2021

Reporting Summary

Nature Research wishes to improve the reproducibility of the work that we publish. This form provides structure for consistency and transparency in reporting. For further information on Nature Research policies, see our Editorial Policies and the Editorial Policy Checklist.

Statistics

For all statistical analyses, confirm that the following items are present in the figure legend, table legend, main text, or Methods section.

n/a	Confirmed	
☐	☒	The exact sample size (*n*) for each experimental group/condition, given as a discrete number and unit of measurement
☐	☒	A statement on whether measurements were taken from distinct samples or whether the same sample was measured repeatedly
☐	☒	The statistical test(s) used AND whether they are one- or two-sided<br>*Only common tests should be described solely by name; describe more complex techniques in the Methods section.*
☒	☐	A description of all covariates tested
☒	☐	A description of any assumptions or corrections, such as tests of normality and adjustment for multiple comparisons
☒	☐	A full description of the statistical parameters including central tendency (e.g. means) or other basic estimates (e.g. regression coefficient) AND variation (e.g. standard deviation) or associated estimates of uncertainty (e.g. confidence intervals)
☒	☐	For null hypothesis testing, the test statistic (e.g. *F*, *t*, *r*) with confidence intervals, effect sizes, degrees of freedom and *P* value noted<br>*Give P values as exact values whenever suitable.*
☒	☐	For Bayesian analysis, information on the choice of priors and Markov chain Monte Carlo settings
☒	☐	For hierarchical and complex designs, identification of the appropriate level for tests and full reporting of outcomes
☒	☐	Estimates of effect sizes (e.g. Cohen's *d*, Pearson's *r*), indicating how they were calculated

Our web collection on statistics for biologists contains articles on many of the points above.

Software and code

Policy information about availability of computer code

Data collection	TIRF experiments: cellSens Dimensions software (version 1.18, Olympus)<br>Optical trap experiments: Bluelake software (version b11, Lumicks)
Data analysis	TIRF experiments: ImageJ (version 2.0.0-rc-69/1.52p)<br>Optical trap experiments: custom Matlab (MathWorks) scripts

For manuscripts utilizing custom algorithms or software that are central to the research but not yet described in published literature, software must be made available to editors and reviewers. We strongly encourage code deposition in a community repository (e.g. GitHub). See the Nature Research guidelines for submitting code & software for further information.

Data

Policy information about availability of data

All manuscripts must include a data availability statement. This statement should provide the following information, where applicable:

- Accession codes, unique identifiers, or web links for publicly available datasets
- A list of figures that have associated raw data
- A description of any restrictions on data availability

The data that support our findings are available under this link: https://data.goettingen-research-online.de/dataverse/schaedel_lorenz_2021

Field-specific reporting

Please select the one below that is the best fit for your research. If you are not sure, read the appropriate sections before making your selection.

- [x] Life sciences
- [ ] Behavioural & social sciences
- [ ] Ecological, evolutionary & environmental sciences

For a reference copy of the document with all sections, see nature.com/documents/nr-reporting-summary-flat.pdf

Life sciences study design

All studies must disclose on these points even when the disclosure is negative.

Sample size	For TIRF experiments, data were recorded until at least 50 min of total depolymerization time and 800 min of polymerization time per condition were reached in order to achieve good estimations of the catastrophe and rescue frequencies, respectively. For the more challenging optical trap experiments, we decided to measure interactions between at least 30 filament pairs per condition, thus ensuring reasonable sample sizes for robust comparison between conditions. We set these target sample sizes based on our previous experience with similar systems.
Data exclusions	Before starting the experiments, the following criteria were established for including data:<br>For TIRF experiments, only microtubules were included that were not visibly stuck to the surface nor grew against an obstacle from the analysis.<br>For optical trap experiments, only filament pairs, where the intermediate filament was not visibly in contact with a biotinylated region on the microtubule were included in the analysis.
Replication	Experiments were performed at least three times on different days (all replications were successful).
Randomization	We measured samples with and without vimentin interchangeable by TIRF microscopy to exclude any time-depending effects. We could not apply further randomization to our study, as our study does not include humans and we did not expect any significant difference between our protein samples used for the experiments.
Blinding	No other researchers than the authors who recorded the data were involved in the study. Therefore, the authors also had to be responsible for the analysis and blinding was not possible.

Reporting for specific materials, systems and methods

We require information from authors about some types of materials, experimental systems and methods used in many studies. Here, indicate whether each material, system or method listed is relevant to your study. If you are not sure if a list item applies to your research, read the appropriate section before selecting a response.

Materials & experimental systems

n/a	Involved in the study	
x		Antibodies
x		Eukaryotic cell lines
x		Palaeontology and archaeology
x		Animals and other organisms
x		Human research participants
x		Clinical data
x		Dual use research of concern

Methods

n/a	Involved in the study	
x		ChIP-seq
x		Flow cytometry
x		MRI-based neuroimaging
